# Comprehensive genomic profiling of over 10,000 advanced solid tumors

**DOI:** 10.18632/oncotarget.28757

**Published:** 2025-07-25

**Authors:** Jean-Paul De La O, Jess R. Hoag, Angela K. Deem, Min Wang, Arthur Starodynov, Sameer S. Udhane, Janine R. LoBello, Nishitha Therala, David W. Hall, Gargi D. Basu, Frederick L. Baehner

**Affiliations:** ^1^Exact Sciences Corporation, Madison, WI 53719, USA

**Keywords:** solid tumors, comprehensive genomic profiling, matched therapy, gene fusions, limit of detection

## Abstract

Purpose: To summarize clinically relevant genomic alterations in solid tumor samples from over 10,000 patients.

Methods: Descriptive statistics were used to summarize findings of retrospectively analyzed OncoExTra assay data from solid tumor samples.

Results: The analysis cohort included 11,091 solid tumor samples from 10,768 patients. Therapeutically actionable alterations were present in 92.0% of patient samples. Biomarkers associated with on- or off-label FDA-approved therapies were detected in 29.2% and 28.0% of samples, respectively. The prevalence of hotspot alterations detected at variant allele frequency (VAF) <5% was analyzed among 7,481 samples (67.5%) harboring ≥1 of these events: 13.7% (1,022 of 7,481) had ≥1 alteration detected at VAF <5%, and 9.8% (558 of 5,690) of hotspot alterations associated with an on- or off-label FDA-approved therapy were detected at VAF <5%. Common and rare mutations in the *TERT* promoter were found in 8.4% (933) of samples. Whole transcriptome sequencing detected clinically relevant fusions in 7.5% of samples, with highest frequencies in prostate cancer (42.0%). The *METe14* transcript was found in 14 NSCLC samples (2.7%).

Conclusions: The broad capabilities of the OncoExTra assay detected therapeutically actionable and other clinically relevant genomic events that can inform clinical decision-making for patients with advanced solid tumors.

## INTRODUCTION

Precision medicine in oncology is highly dynamic, and regulatory approvals for cancer therapies targeting genomic aberrations within defined patient subpopulations continue to increase [1]. Between 2015 and 2021, at least 25% of drugs approved by US FDA were biomarker-matched therapies [2], and over 40% of all oncology drugs approved between 1998 and 2022 were for precision oncology [3, 4]. Tumor genomics and functional studies have linked a range of genetic alterations, including single nucleotide variants (SNVs), insertions and deletions (indels), copy number alterations (CNAs), and gene fusions, with drug-response phenotypes. The field has also seen an increase in tumor-agnostic therapy approvals linked to predictive biomarkers, including *NTRK1/2/3* and *RET* fusions, *BRAF* V600E mutations, as well as genomic signatures for microsatellite instability (MSI) and tumor mutational burden (TMB) to identify patients who may benefit from immunotherapies. The expansion of validated biomarkers and precision oncology drugs is driving an increase in the proportion of patients with tumors harboring a biomarker predictive of therapy response [4], and evidence continues to accumulate in support of personalized therapies resulting in better clinical outcomes [5–16]. Thus, it is increasingly important to test for these predictive biomarkers to inform and support oncologists and patients in selecting the most appropriate therapy options amidst the rapidly expanding landscape of precision cancer therapies to maximize patient outcomes.

As the number of actionable alterations increases, comprehensive genomic profiling (CGP) assays employing next generation sequencing (NGS) are becoming more widely utilized in patients with solid tumors. Multi-gene panel tests that probe DNA alterations in 200–1,000 genes or across the whole exome (approximately 20,000 genes) are increasingly prioritized over single-gene or small-panel assays. This reflects both the growing acknowledgement by clinicians regarding their clinical utility and clinical practice guidelines that support use of CGP across all solid tumors [17–19]. The choice of CGP is also future-proof, providing data that are readily available for clinicians if a new alteration with a matched therapy becomes available during a patient’s course of treatment. Further, in contrast to small-panel assays, CGP assays can identify genomic signatures such as TMB and MSI that are associated with FDA-approved tumor-agnostic immunotherapy drugs [18], underscoring the clinical utility of using broad, multimarker tumor panels. Importantly, there is increasing reimbursement associated with CGP by healthcare payers [20, 21].

Commercially available tumor profiling assays are distinguished by several characteristics. The breadth of genome sequencing varies widely among tests. Smaller-panel testing can miss alterations by focusing on a relatively narrow list of hotspot alterations, which can result in the need for additional genomic testing that delays treatment decisions and risks running out of tumor tissue for analysis. However, increased sequencing breadth often results in decreased sequencing depth and a higher (i.e., poorer) limit of detection for variants [22, 23]. The focus on capturing a greater number of variants may thus come with the risk of missing rare, low frequency, variants, which may be present because of subclonal alterations in a heterogeneous tumor sample or low purity of a sample. The ability to discriminate germline alterations is another feature of some assays. Assays that only annotate alterations in tumor specimens can inaccurately identify germline alterations as somatic alterations. In contrast, assays that sequence both matched tumor and normal samples can discriminate germline from somatic alterations, opening up the opportunity to refer patients with germline oncogenic alterations for genetic counseling and preventing overestimation of TMB [18, 24–27]. Tests can also integrate multiple NGS platforms to simultaneously sequence DNA and RNA, and these tests have improved ability to detect alterations—particularly fusions—as well as the capability of identifying cancer-relevant alternate RNA transcripts [28–30]. Tumor profiling tests may also offer immunohistochemistry (IHC) testing to characterize expression of biomarkers such as HER2, PD-L1 and mismatch repair (MMR) proteins, which can identify patients who may benefit from anti-HER2 and immune-based therapies. Ultimately, as the precision medicine landscape has evolved, so too has biomarker testing, making it critically important to understand the advantages and limitations of available testing options for the identification of relevant biomarkers for patients with solid tumors.

The OncoExTra^®^ assay (formerly GEM ExTra) is a whole exome, whole transcriptome, tumor-normal genomic profiling assay that is designed to identify somatic (tumor-specific) SNVs, CNAs, indels, gene fusions, and alternative transcripts. The assay also determines MSI status and TMB. This retrospective study aimed to summarize clinically relevant results from comprehensive genomic profiling of advanced solid tumor samples from more than 10,000 patients tested with the OncoExTra assay.

## RESULTS

### Sample characteristics

A total of 11,091 solid tumor samples from 10,768 patients were included in the analysis population. Most (60.3%) samples were collected from female patients, and the median age of all patients was 62 years (interquartile range, 51–70 years). Therapeutically actionable alterations were present in 92.0% of patient samples. Biomarkers associated with on-label matched therapies were detected in 29.2% of samples, and biomarkers associated with off-label matched therapies were detected in 28.0% of samples (Table 1).

**Table 1:** Sample characteristics

Characteristic	
**Patients, *n***	10,768
**Patient samples, *n***	11,091
**Samples per patient, *n* (%)**	
1 sample	10,468 (97.2%)
2 samples	280 (2.6%)
≥3 samples	20 (0.2%)
**Age (years) at sample collection, median (IQR)**	62 (51.0, 70.0)
≤50 years	2615 (23.6%)
>50 years	8432 (76.0%)
Missing	44 (0.4%)
**Sex, *n* (%)**	
Male	4,403 (39.7%)
Female	6,687 (60.3%)
Missing	1 (<0.01%)
**Therapeutic actionability, *n* (%)**	
on-label biomarker^*^	3,240 (29.2%)
off-label biomarker^*^	3,110 (28.0%)
Any therapeutically actionable biomarker	10,206 (92.0%)

^*^ *n* = 1,172 samples had both on- and off-label biomarkers.

**Figure 1:**
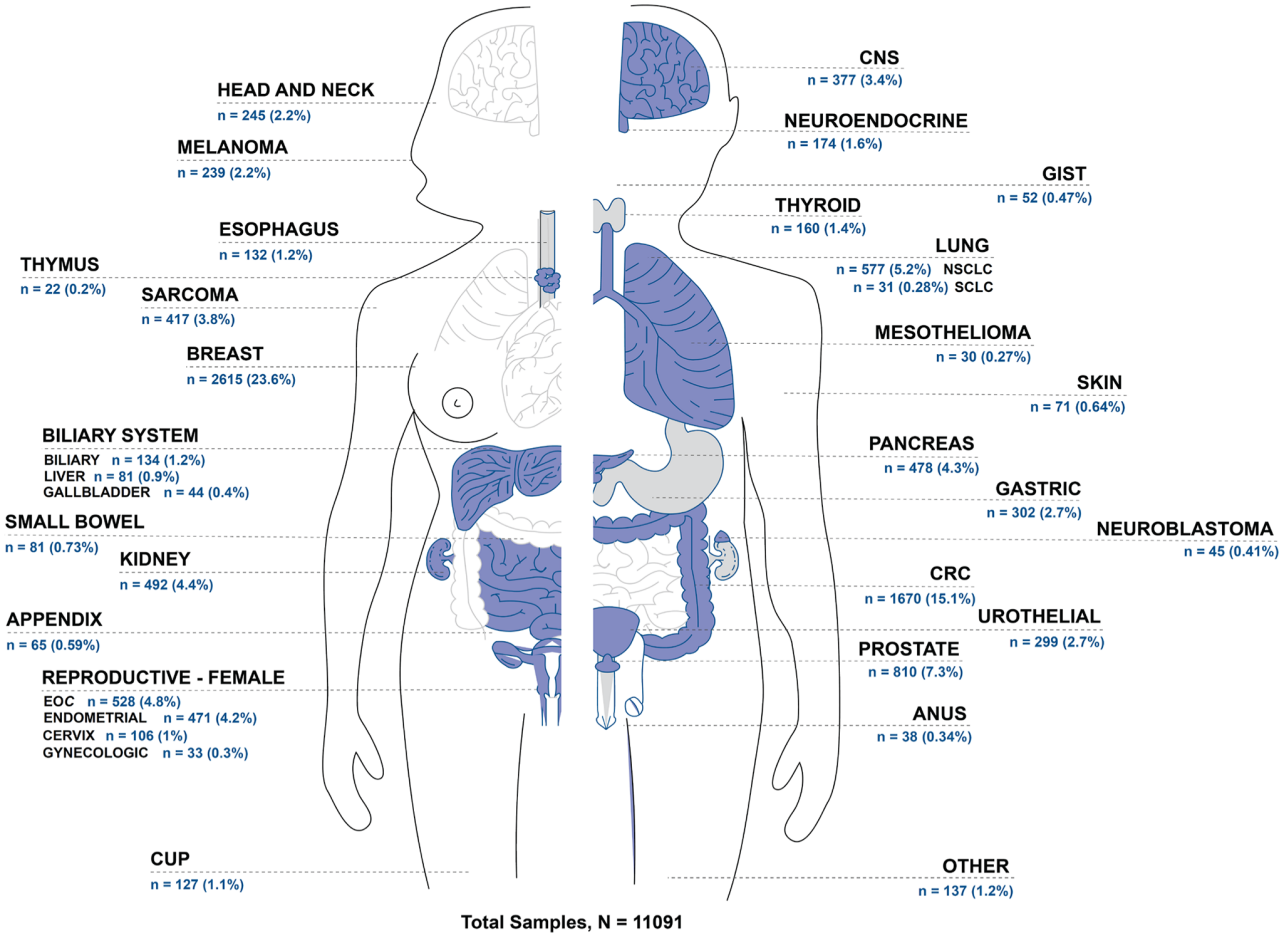
Distribution of samples by tumor type.

Across 31 distinct tumor types represented in the analysis cohort, the five most common tumor types were breast cancer, colorectal cancer (CRC), prostate cancer, non-small cell lung cancer (NSCLC), and epithelial ovarian cancer (EOC), which collectively accounted for 56% of all samples (Figure 1). The analysis cohort also included samples from cancers of unknown primary (CUP) as well as samples from more rare tumor types, classified as “Other”.

### Landscape of actionable alterations

**Figure 2:**
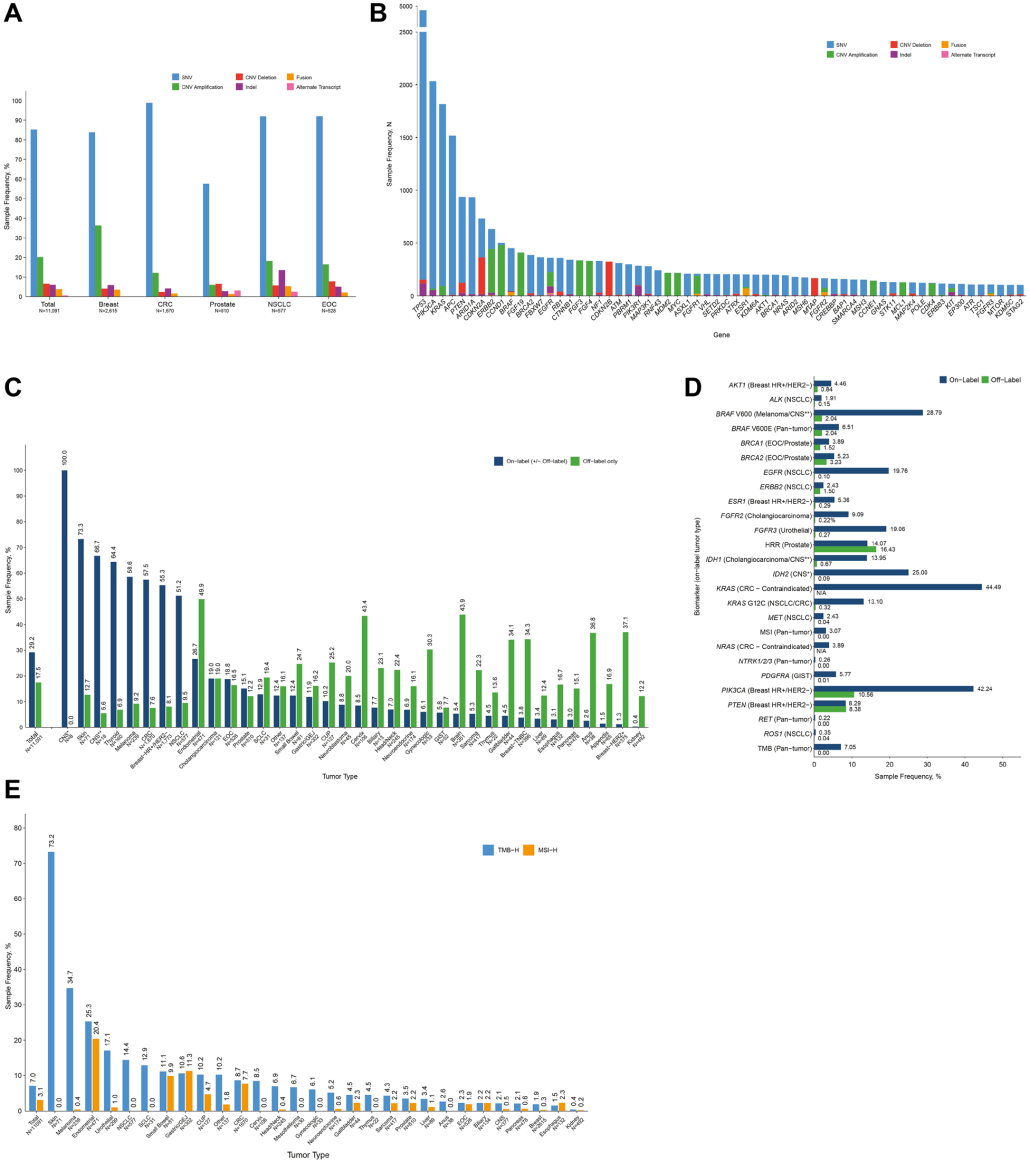
Distribution of samples with therapeutically actionable alterations in the OncoExTra analysis cohort. (**A**, **B**) Distribution of samples with therapeutically actionable alterations (A) by alteration type, overall and for the five most common cancer types in the cohort and (B) by alteration type for 61 genes detected in >100 samples. (**C**, **D**) Distribution of samples with biomarkers associated with on-label and off-label therapies (C) overall and by tumor type and (D) by biomarker. CNS* = grade II astrocytoma and oligodendroglioma; CNS** = low-grade glioma (**E**) Distribution of samples characterized by composite biomarkers TMB-high and MSI-high, overall and by tumor type. Data labels indicate the percentage of samples with TMB-high and MSI-high in each cancer type. For (A–D), samples can have >1 alteration as well as >1 alteration within the same gene.

Single nucleotide variants were the most frequently observed alteration type among therapeutically actionable alterations, present in 85.3% of samples (Figure 2A and Supplementary Table 1). Copy number variant amplifications and deletions were present in 20.2% of samples and 6.6% of samples, respectively. Compared to SNVs and CNVs, indels, gene fusions, and alternative transcripts were present at lower frequencies (6.1%, 3.9%, and 0.6%, respectively) (Figure 2A). The distribution of alteration types among samples with actionable alterations was generally consistent across the five most common cancer types (Figure 2A); however, SNVs were less prevalent in prostate cancer, CNV amplifications were more prevalent in breast cancer, and indels were more prevalent in NSCLC. The distribution of alteration types for 61 genes with actionable alterations present in at least 100 samples is shown in Figure 2B and further detailed in Supplementary Table 2.

Actionable alterations were further characterized based on associations with on- or off-label FDA-approved matched therapies, summarized by tumor type (Figure 2C) and by biomarker (Figure 2D). The frequency of biomarkers associated with on-label therapies varied by tumor type. Several cancers with relatively low percentages of biomarkers associated with on-label therapies had relatively high levels of biomarkers associated with off-label therapies. For example, the central nervous system (CNS) tumor samples had low frequencies of four different biomarkers approved in all solid tumor indications (5.4% collectively across *BRAF* V600E, *NTRK2* fusion, TMB-high, and MSI-high), whereas the frequency of biomarkers associated with off-label FDA-approved matched therapies was much higher (43.9%). Similarly, some gene alterations with matched therapies occurred at relatively higher frequencies in on-label cancer types, including alterations in *PIK3CA* (42.2% in on-label HR+/HER2- breast cancer vs. 10.6% in off-label cancers) and in *EGFR* (19.8% in on-label NSCLC vs. 0.1% in off-label cancers) (Figure 2D). Both MSI-high and TMB-high contributed to the overall frequency of on-label indications and were responsible for the majority of on-label alterations in some cancer types (Figure 2E).

### Alterations detected at low variant allele frequency

**Figure 3:**
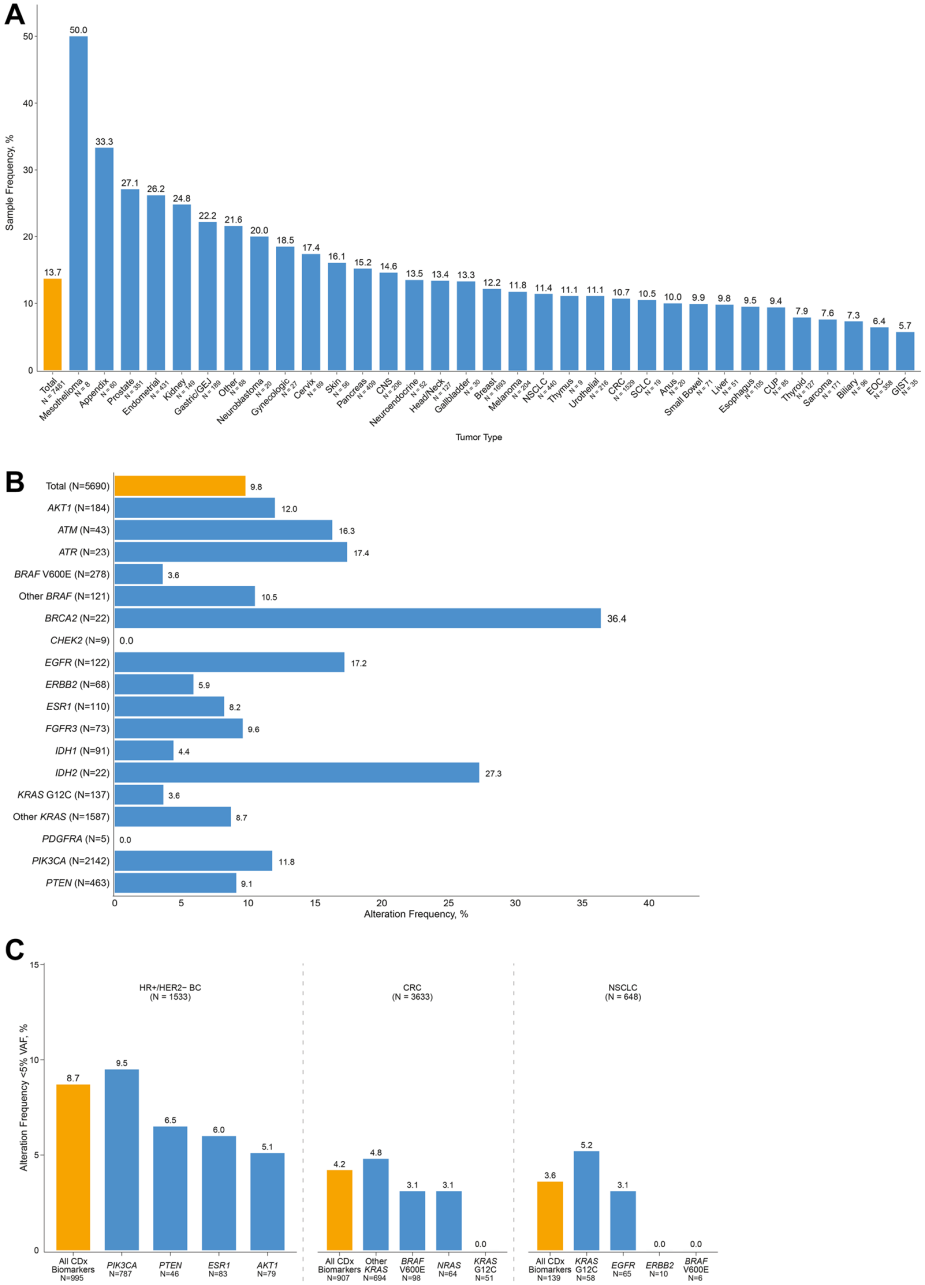
Hotspot alterations detected at low VAF. (**A**) Distribution of samples with at least one hotspot alteration detected at a VAF <5%, overall and by cancer type. (**B**) Distribution of hotspot biomarker alterations associated with on- and off-label matched therapies detected at a VAF <5% by biomarker. (**C**) Distribution of hotspot biomarker alterations associated with on-label matched therapies detected at a VAF <5% for HR+/HER2- breast cancer, CRC, and NSCLC.

Across the entire exome, the OncoExTra assay is validated to detect alterations above 5% VAF with high sensitivity; however, for a subset of clinically important hotspot alterations, the assay is validated to detect alterations at VAFs above 1%. Therefore, we examined the potential clinical impact of alterations detected at low VAF by analyzing variant calls at hotspots validated for this lower LOD. A total of 67.5% (7,481 of 11,091) of samples had at least one hotspot alteration detected, representing 12,954 variant records across the subset of validated hotspot alterations. Among this subset, 13.7% (1,022 of 7,481) of samples had at least one alteration detected at a VAF <5% (Figure 3A), and 10.1% (1,313 of 12,954) of all detected hotspot alterations were detected at a VAF <5%.

When considering hotspot alterations associated with FDA-approved therapies, 9.8% (558 of 5,690) of detected hotspot alterations associated with an on- or off-label FDA-approved matched therapy were detected at VAF <5%. The distribution of alterations associated with on- and off-label matched therapies detected at a VAF <5% is shown in Figure 3B.

Three cancer types had more than 100 samples with at least one hotspot alteration detected that is associated with an on-label or off-label FDA-approved therapy: breast cancer, CRC, and NSCLC. In HR+/HER2- breast cancer, 8.7% (87 of 995) of hotspot alterations associated with an on-label matched therapy were detected at a VAF <5%, comprising alterations in *AKT1*, *ESR1*, *PIK3CA*, and *PTEN* (Figure 3C). In CRC, 4.2% (38 of 907) of hotspot alterations associated with on-label matched therapies, which included *BRAF* V600E, *KRAS* G12C, other *KRAS* alterations (contraindicated), and *NRAS* alterations (contraindicated), were detected at a VAF <5%. In NSCLC, 3.6% (5 of 139) of hotspot alterations associated with on-label matched therapies, which included *BRAF* V600E, *EGFR* alterations, *ERBB2* alterations, and *KRAS* G12C, were detected at a VAF <5%.

### Clinically relevant alterations detected by whole transcriptome sequencing

**Figure 4:**
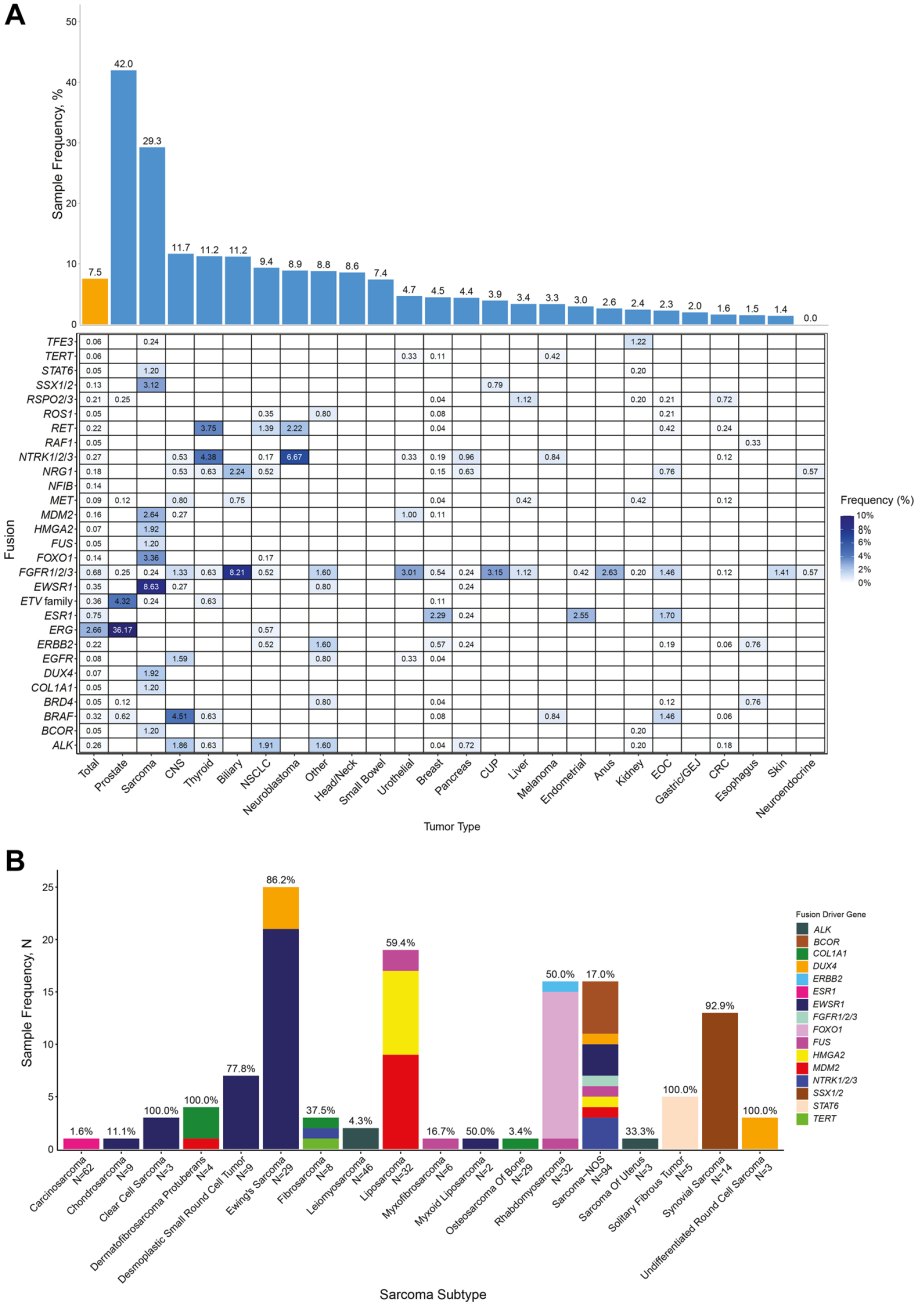
Characterization of clinically relevant fusions detected by WTS in solid tumor samples. (**A**) Frequency of clinically relevant fusion drivers detected in samples, overall, by tumor type and by partner genes forming fusions. (**B**) Frequency of fusions and partner genes detected in sarcoma subtypes. Number of fusions with indicated oncogenic driver gene in each tumor type.

Whole transcriptome sequencing (WTS) allowed fusion and alternative transcript detection in tumor samples. Overall, WTS detected clinically relevant fusions in a total of 7.5% of samples (Figure 4A). As expected, WTS was more sensitive than whole exome sequencing (WES) for fusion detection, with 30.9% of fusions approved as companion diagnostic biomarkers for matched therapies in solid tumors found in the transcriptomic data only. The distribution of clinically relevant fusions varied across tumor types, and the highest frequencies of fusions were detected in prostate cancer (42.0%) and sarcoma (29.3%) (Figure 4A). Owing to its prevalence in prostate cancer (293 of 810 samples (36.2%)), *ERG* was the most frequent fusion driver gene identified in our cohort (295 of 11,091 samples (2.7%)) (Figure 4A). Of note, *ESR1* fusions were observed not only in breast cancer (2.3%) but also in endometrial cancer (2.6%) and EOC (1.7%).

In sarcomas, in addition to having prognostic and therapeutic implications, fusions serve as key diagnostic biomarkers that can refine disease diagnosis beyond histological examination. Further, about 40% of sarcoma subtypes are driven by more than 100 different fusion proteins [31]. Therefore, we evaluated the presence of fusions with diagnostic prognostic, and/or therapeutic relevance within sarcoma subtypes (Figure 4B). We identified a high frequency of fusions in Ewing’s sarcoma, where 86.2% of samples contained fusions (25 of 29 samples), including 21 samples (72.4%) harboring an *EWSR1* fusion and 4 samples (13.8%) harboring a *DUX4* fusion. The frequency of fusions was also high in desmoplastic small round cell tumors (77.8%; 7 of 9 samples) and in dermatofibrosarcoma protuberans (100%; 4 of 4 samples), driven by *EWSR1* or *COL1A1* fusions, respectively. Pathognomonic fusions were also identified, such as *PAX3*-*FOXO1* and *PAX7*-*FOXO1* in alveolar rhabdomyosarcoma, *SS18*-*SSX* in synovial sarcoma, and *NAB2*-*STAT6* in solitary fibrous tumors. Rare *NTRK1/2/3* fusions were identified in two sarcoma subtypes, suggesting NTRK-directed therapies might be of benefit in these cases. All undifferentiated round cell sarcomas harbored *DUX4* fusions (100%; 3 of 3 samples). A *TERT* fusion, likely associated with increased *hTERT* expression and with poor patient outcome [32], was identified in a fibrosarcoma sample. Collectively, we find that WTS with the OncoExtra assay identifies clinically relevant fusion constructs to guide therapy selection and aid in accurate diagnosis and prognostication in sarcomas.

The frequency of five clinically relevant alternate transcripts, including androgen receptor variant 7 (*ARv7*), hepatocyte growth factor receptor exon 14 skip (*METe14*), and epidermal growth factor receptor variants (*EGFRv*) vIII/vIVa/vIVb, was determined overall and by cancer type. At least one alternate transcript was found in 1% of samples (100 of 10,227 samples) (Supplementary Table 3). This included 14 NSCLC samples (2.7%) that harbored the *METe14* transcript, which can be targeted with various tyrosine kinase inhibitor (TKI) therapies.

### Alterations in cancer pathways

**Figure 5:**
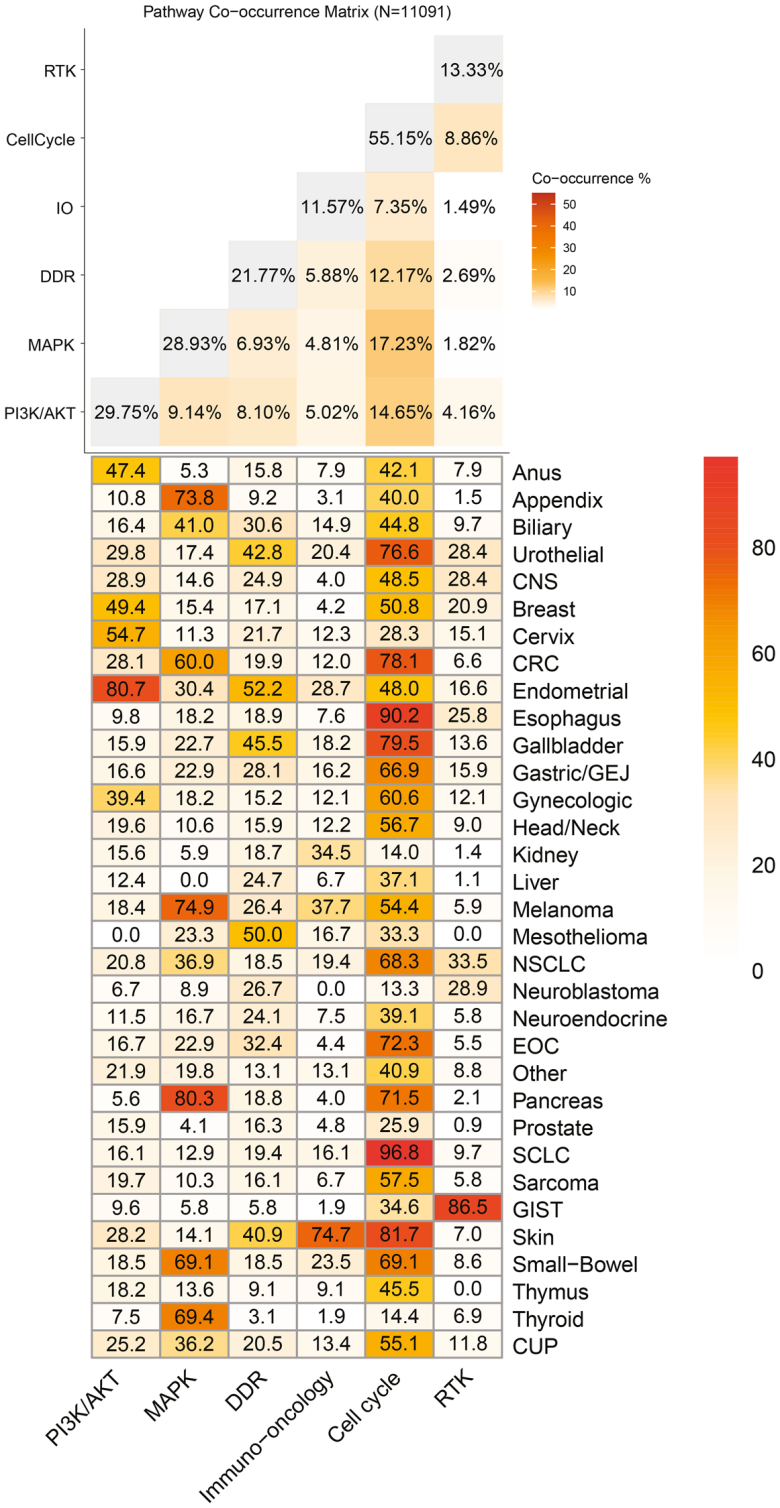
Distribution of cancer-relevant pathways, overall and by tumor type. Top: Co-occurrence matrix of cancer-relevant pathways. Data labels indicate frequency of alterations in a gene within the indicated pathway that is associated with matched therapy. Bottom: Heatmap displaying frequency of alterations in a gene within the indicated pathway that is associated with a matched therapy by cancer type.

We also assessed the frequency of alterations in genes in six cancer-relevant pathways (Supplemental Table 1). The frequency of alterations in these pathways varied greatly across cancer types, from 0% to 96.8% (Figure 5). In some cancer types, including CRC, endometrial cancer, melanoma, pancreas cancer, skin cancer, and small-bowel cancer, alteration frequencies were greater than 50% in more than one pathway (Figure 5). Alterations in cell cycle genes were present in the highest proportion of samples (55.2%; 6,121 of 11,091 samples), driven by eight cancer types for which >70% of samples had at least one altered cell cycle gene: pancreas (71.5%), EOC (72.3%), urothelial (76.6%), CRC (78.1%), gallbladder (79.5%), skin (81.7%), esophagus (90.2%), and SCLC (96.8%). PI3K/AKT/mTOR alterations were detected in 29.8% of samples, with expectedly high frequencies in breast (49.4%) and gynecologic (endometrial, 80.7%; cervix, 54.7%; and gynecologic, 89.4%) cancers. Alterations were found in the MAPK pathway in 28.9% of samples and at high frequency in several gastrointestinal cancers (pancreas, 80.3%; appendix, 73.8%; small-bowel, 69.1%; and CRC, 60.0%) as well as in melanoma (74.9%) and thyroid cancer (69.4%). Overall, 21.8% of samples had an alteration in a DNA damage response (DDR) pathway gene, and the highest frequencies occurred in endometrial cancer (52.2%), mesothelioma (50.0%), and gallbladder cancer (45.5%). A very high proportion of GIST samples harbored alterations in the RTK pathway (86.5%), but the frequency of RTK pathway alterations in the overall cohort was much lower (13.3%). Similarly, alterations in immuno-oncology pathway genes were detected in 74.7% of skin cancer samples, but the frequency of these alterations in the analysis cohort overall was only 11.6%. Notably, many samples had co-occurring alterations in two cancer-relevant pathways, including 17.2% and 14.7% of samples with a cell cycle pathway alteration as well as a MAPK or PI3K/AKT/mTOR pathway alteration, respectively.

### *TERT* alterations

**Figure 6:**
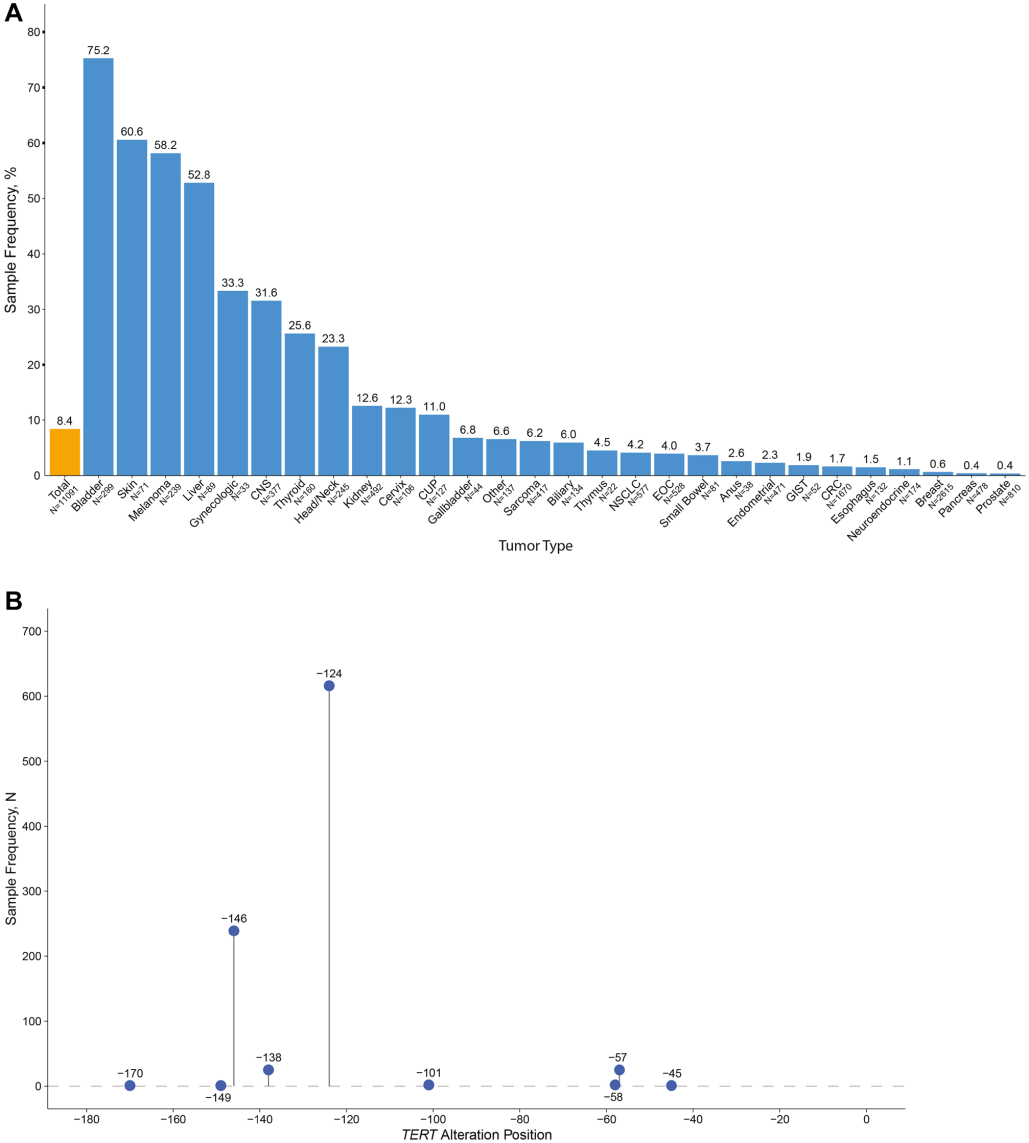
*TERT* promoter mutations. (**A**) Distribution of *TERT* promoter mutations, overall and by tumor type. (**B**) Location and frequency of *TERT* promoter mutations across all tumor types. In addition to alterations at the sites shown, seven *TERT* fusions and eight *TERT* amplifications were also observed.

Mutations in the *TERT* promoter have been shown to upregulate telomerase expression, resulting in aggressive tumor characteristics [33, 34]. Overall, 8.4% (933) of samples had a mutation in the *TERT* promoter, and the frequency varied widely among tumor types (Figure 6A). Most (877 of 946, 92.7%) *TERT* promoter mutations occurred at positions −124 (616 of 638 were C→T) and −146 (239 of 240 were C→T), though alterations at other sites were also observed (Figure 6B). *TERT* promoter mutations were most frequent in bladder-urothelial cancer (75.3%), while skin cancer (60.6%), melanoma (58.2%), and liver cancer (52.8%) also had expectedly high frequencies. *TERT* promoter mutations were detected in 31.6% of CNS tumor samples, consistent with previous reports. We also detected rare *TERT* fusions (7 of 11,091 samples (0.06%)) and *TERT* amplifications (8 of 11,091 samples (0.07%)), which are associated with increased TERT expression and aggressive disease [32].

## DISCUSSION

Comprehensive genomic profiling has been a driving force in the personalized medicine paradigm shift in cancer therapy selection, wherein biomarker analysis and gene-directed therapies are often prioritized over treatments traditionally selected based on tumor histology [35, 36]. Multiple clinical guidelines from professional organizations, including the recent ASCO Provisional Clinical Opinion, recommend CGP for the management of patients with newly diagnosed advanced cancer, relapsed or recurrent disease, or cancer that is refractory to treatment [17–19]. The OncoExTra matched tumor-normal assay provides WES and WTS data in line with these practice guidelines for detecting SNVs, CNVs, indels, gene fusions, composite biomarker signatures, and alternative transcripts in advanced solid tumors [37, 38]. In this study, we retrospectively analyzed the landscape of clinically relevant genomic biomarkers identified by the OncoExTra assay in advanced solid tumor samples collected from more than 10,000 patients, identifying alterations that can be used to select approved matched therapies, for diagnostic and prognostic refinements, or to determine clinical trial eligibility. The combination of expanding approvals of biomarker-directed therapies, clinical trials incorporating predictive biomarkers, and the broad capabilities of the OncoExTra assay resulted in 92% of patients in our cohort being informed of a therapeutically actionable result.

The trade-off between sequencing breadth versus depth is an important consideration in the selection of tumor profiling assays [22, 23]. The OncoExTra assay analyzes the entire exome and has double coverage of cancer-relevant genes, which boosts sequencing depth to ≥800x, allowing alterations with VAFs as low as 5% to be called exome-wide and a subset of clinically significant hotspot SNVs and indels to be called with VAFs above 1%. Here, the OncoExTra assay identified 14% more alterations compared to an assay with an LOD of 5% VAF, and 9.2% (1,022 of 11,091) of samples had a clinically relevant hotspot alteration called at a VAF <5%. CGP assays with increased sensitivity and low LOD may have clinical benefit particularly for patients with advanced cancer who develop subclonal resistance mutations, which is highly relevant for a large proportion of patients with advanced tumors who have undergone multiple lines of prior therapy.

In line with incidence rates in the US, the most common cancer types in the analysis cohort were breast (23.6%), CRC (15.1%), prostate (7.3%), and NSCLC (5.2%) [39]. We identified therapeutically actionable biomarkers in over 50% of these samples, and at frequencies over 80% for breast cancer, CRC, and NSCLC. In metastatic HR+/HER2- breast cancer that has progressed after adjuvant endocrine therapy, a significant progression-free survival (PFS) benefit can be achieved in the context of PI3K/AKT pathway mutations when alpelesib [40], capivasertib [41], or inavolisib [42] is administered with fulvestrant (alpelesib and capivasertib) or with fulvestrant plus palbociclib (inavolisib). Further, when HR+/HER2- breast cancer patients progress on endocrine therapy due to emergence of *ESR1* resistance mutations, elacestrant therapy can significantly prolong PFS [43]. Recently, therapies targeting constitutive KRAS activation were shown to extend PFS for patients with NSCLC and CRC harboring *KRAS* G12C mutations, including adagrasib (CRC and NSCLC) [44, 45] and sotorasib (NSCLC) [46]. The *KRAS* G12C mutation is present in ~25% of NSCLCs [47, 48], but is less common in CRC (3–4%) [49]; however, patients with *KRAS* G12C-mutated CRC have a poor prognosis [50, 51], and drugs to quench RAS signaling offer a new therapeutic modality. [52, 53]. Likewise, in CRC, *KRAS* and *NRAS* alterations are important biomarkers for predicting resistance to monotherapy with anti-EGFR antibodies [54]. Finally, approximately 25% of prostate cancers harbor DDR alterations, conferring sensitivity to PARP inhibitors. Patients with DDR-deficient metastatic prostate cancer may qualify for treatment with olaparib [55], rucaparib [56], talazoparib plus enzalutamide [57, 58], or niraparib plus abiraterone [59], which can have higher objective response rates and PFS compared to androgen antagonist drugs alone, although there is some variation in response depending on which DDR gene is altered [60, 61]. Of relevance for patients who received the OncoExtra assay, these agents were shown to be effective in advanced disease after prior therapy.

The OncoExTra assay simultaneously assesses TMB and MSI status, making it an efficient method to detect therapeutically relevant alterations across cancer types. Testing for MSI status and TMB is indicated for all patients with advanced or metastatic solid tumors, potentially qualifying them for immunotherapy [18]. Two features of the OncoExTra assay contribute to accurate assessment of TMB. First, WES more comprehensively and efficiently assesses alterations genome-wide compared with smaller panels. Notably, current ASCO guidelines note high variability among panel-based sequencing tests in TMB calculation and recommend WES for TMB testing [18]. Second, germline-alteration subtraction avoids inaccurate calling of germline alterations as somatic events, leading to TMB overestimation, especially for patients of non-European ancestry due to the composition of established databases currently used for germline subtraction by tumor-only tests [27, 62].

Clinical guidelines support RNA sequencing analysis to identify fusions due to the superior detection rate compared to DNA sequencing [18]. In this cohort overall, 7.5% of samples had clinically relevant gene fusions, and 3.9% of these were therapeutically actionable. Similar to other reports [29, 30, 63, 64], fusions were more likely to be identified by WTS with the OncoExTra assay, and 30.9% of fusions associated with matched therapies were identified by WTS only (i.e., were not detected in the WES data).

Among fusion events, *ERG* was the most frequent gene partner (2.7% overall), driven by its high prevalence in prostate cancer (36.2%). Previous studies reported that prostate cancers harboring *TMPRSS2-ERG* fusions are more dependent on androgen signaling and may be more responsive to androgen deprivation therapy [65]. Our assay also reported *FGFR1/2/3* fusions in 0.68% of samples, though prevalence was higher in biliary (8.21%) and urothelial (3.01%) cancer samples. While rare in most cancers, these fusions indicate poor prognosis and are candidates for FDA-approved FGFR-targeted therapies, including infigratinib, pemigratinib and futibatinib for cholangiocarcinoma with *FGFR2* fusions and erdaftinib for urothelial cancers with *FGFR3* fusions [66]. Importantly, it has been demonstrated that patients treated with a matched therapy targeting a gene fusion have both improved response rates compared to patients treated with an unmatched therapy, as well as improved response rates compared with patients who received a matched therapy for a non-fusion alteration [67]. Consistent with excellent therapeutic benefit of targeting driver gene fusion events, in December 2024, FDA approved zenocutuzumab-zbco for refractory pancreatic cancer and NSCLC that are *NRG1*-fusion-positive; the data cut-off for the present study occurred before this approval, but 3 events each were observed in our pancreatic cancer (0.63%) and NSCLC (0.52%) cohorts.

Molecular characterization of tumor samples, including NGS analysis, is routinely used to diagnose sarcomas, where diagnosis and classification can be challenging using immunohistochemistry alone due to overlapping morphological manifestations [68]. Sarcoma formation can be driven by transcriptional dysregulation caused by gene rearrangements leading to fusion protein formation [31], and 44 specific fusion genes are diagnostic for select sarcoma subtypes [69]. Our assay reported several diagnostic fusions, including *PAX3* and *PAX7* joining with *FOXO1* in alveolar rhabdomyosarcoma and *SS18-SSX* fusion in synovial sarcomas. Certain fusions are associated with an aggressive clinical course and inferior overall survival, such as *CIC-DUX4* fusion [70], which was reported in Ewing’s sarcoma as well as undifferentiated round cell sarcoma in our cohort. We also reported *TNS1*-*ALK* fusions in two uterine sarcomas, and a case report described a patient with leiomyosarcoma benefiting from matched therapy with the ALK inhibitor brigatinib after failing multiple lines of chemotherapy [71]. Our cohort also included five sarcoma samples harboring an *NTRK1* or *NTRK3* fusion, which is predictive of response to the FDA-approved drugs entrectinib, larotrectinib, and repotrectinib. Thus, transcriptome sequencing-based fusion detection may complement current histology-based classification as well as inform drug selection and clinical trial eligibility for sarcomas.

*TERT* promoter mutations have been reported in at least 50 cancer types and are associated with aggressive disease and poor clinical outcomes [33, 34]. The OncoExTra assay identified both common and rare *TERT* promoter alterations, which are prognostic for cancer progression, including commonly occurring G-to-A substitutions occurring –124 and –146 bp relative to the *TERT* transcription start site known to drive increased telomerase expression [34, 72, 73]. Additionally, *TERT* amplifications and fusion events were observed in a small number of samples (<1% for both events), which are associated with increased *TERT* expression and worse prognosis [32]. Notably, the first telomerase-targeting drug (imetelstat) was approved in June 2024 to treat patients with lower-risk myelodysplastic syndromes who did not respond to erythropoiesis-stimulating drugs [74], and there remains great interest in expanding therapeutic opportunities to intervene in telomerase-activated tumors.

An important limitation of this study is the lack of clinical outcomes data. Other studies have reported improved outcomes for patients treated with a genomically matched therapy [5–16], and our retrospective analysis of a large patient cohort confirms that the OncoExTra assay detected alterations in biomarkers associated with clinically available matched therapies could support therapy selection or identify clinical trials. Similarly, our cohort was not preselected based on any prior therapy, and adaptive resistance mechanisms can influence the prevalence of several critical genomic biomarkers. In addition, in this analysis, we combine alterations from primary or metastatic sites based on the primary tumor diagnosis, although it is recognized that, at least for some cancer types, the landscape of genetic alterations is different in primary compared to metastatic lesions. Finally, as expected, fusion detection using WTS data was more sensitive compared to using DNA data. DNA-based calling algorithms are known to have limitations in detecting fusions as genomic breakpoints occur at random locations, and DNA-based sequencing needs to cover introns to be more efficient at reporting fusions [28, 29].

## MATERIALS AND METHODS

### Study population

We retrospectively analyzed OncoExTra assay data from solid tumor samples meeting minimal sample requirements between April 2018 and May 2024. All assays were performed in Exact Sciences’ Clinical Laboratory Improvement Amendments (CLIA)-approved, College of American Pathology (CAP)-certified laboratory facility in Phoenix, Arizona. Patients <18 years of age at the time of sample submission were categorized as <18 years and patients ≥89 years of age were categorized as ≥89 years.

### Comprehensive genomic profiling

Validation of the OncoExtra CGP assay was previously described [37]. The assay requires a solid tumor sample and a matched normal/germline sample, usually obtained from a whole blood sample. DNA extracted from both samples and targeted sequences from DNA libraries were captured using a custom IDT xGen exome capture (Integrated DNA Technologies, Coralville, IA, USA) probe set and sequenced using Illumina NovSeq 6000 (Illumina, San Diego, CA, USA). Tumor DNA was sequenced to ≥400× depth and to 800× depth for 440 cancer-associated genes, and the normal DNA sample was sequenced to ≥180× depth. The assay thus focuses on both breadth, by sequencing the entire exome, and depth by increasing sequencing coverage in known cancer-associated regions. Sequences are aligned to a human reference genome (currently the hs37d5 build), and alterations are identified using a custom bioinformatic pipeline.

For WTS sequencing, RNA is extracted from tumor tissue samples, rRNA is depleted, and the remaining RNA is subsequently reverse transcribed. RNA libraries are sequenced to 100M total reads. Reads are aligned to the human reference genome to detect gene fusions and five alternative transcripts: androgen receptor variant 7 (*ARv7*), hepatocyte growth factor receptor exon 14 skip (*METe14*), and the epidermal growth factor receptor variants *EGFRvIII*, *EGFRvIVa*, and *EGFRvIVb*.

### Classification of therapeutic actionability

An alteration was deemed actionable if any of the following criteria were met: (1) It had an associated FDA-approved therapy in the cancer type in which it was found (i.e., use of the therapy would be on-label) (2) It had an associated FDA-approved therapy in a cancer type that was different from the one in which it was found (i.e., use of the therapy would be off-label); (3) It was in a gene or pathway that was being targeted in an active clinical trial; (4) There was evidence in the literature, or there was mention in clinical guidelines, of possible therapeutic efficacy of a matched therapy for the alteration in any cancer; or (5) It was contraindicated for an FDA-approved therapy in the cancer in which it was found (also considered on-label). We report actionability based on whether an alteration met one of these criteria at the time the sample was processed. In some analyses, we examine a subset of actionability, focusing on alterations with on-label versus off-label matched therapies. For these analyses, we use the FDA-approved therapies as of September 5, 2024.

### Statistical analysis

The primary objectives of this study were to describe the distribution of patient samples across solid tumor types and report the prevalence of actionable biomarkers and gene pathways known to be important in cancer. The prevalence of clinically relevant diagnostic and prognostic biomarkers is also reported. Cancer-relevant pathways included the phosphoinositide 3-kinase/protein kinase B/mammalian target of rapamycin (PI3K/AKT/mTOR), mitogen-activated protein kinase (MAPK), DNA damage response (DDR), immune-oncology, cell cycle, and receptor tyrosine kinase (RTK) pathways (Supplementary Table 4). Descriptive statistics were used to summarize sample characteristics and the landscape of alterations.

All analyses were performed using SAS software (version 9.4, SAS Institute, Cary, NC, USA) and R Statistical Software (version 4.4.0). As this was a descriptive study with no formal hypothesis testing, no statistical power calculations were performed.

## CONCLUSIONS

Cancer is a heterogeneous disease, with clinically important genetic differences among patients, within tumors, and across metastatic sites. Distinct genomic alterations can inform therapy selection, predict drug response, and uncover adaptive mechanisms in refractory disease. Comprehensive molecular characterization with the OncoExTra whole exome, whole transcriptome, matched tumor-normal assay provides patients with results to support clinicians in their efforts to select approved personalized therapies or recommend clinical trials.

## SUPPLEMENTARY MATERIALS



## Abbreviations

ARv7: Androgen receptor variant 7
CGP: Comprehensive genomic profiling
CNA: Copy number alteration
CNS: Central nervous system
CRC: Colorectal cancer
CUP: Cancers of unknown primary
EGFRv: Epithelial growth factor receptor variant
EOC: Epithelial ovarian cancer
Indel: Insertions and deletions
LOD: Limit of detection
METe14: Hepatocyte growth factor receptor exon 14 skip
MSI: Microsatellite instability
NSCLC: Non-small cell lung cancer
NGS: Next-generation sequencing
SNV: Single nucleotide variant
TMB: Tumor mutational burden
VAF: Variant allele frequency
WES: Whole exome sequencing
WTS: Whole transcriptome sequencing
