# Supplementary material for: Comprehensive genomic profiling of over 10,000 advanced solid tumors
Source: Oncotarget. 2025 Jul 25;16:587–603. doi: 10.18632/oncotarget.28757 (PMC12406462; doi:10.18632/oncotarget.28757)
Supplement: Supplementary file 1 [file oncotarget-16-28757-s001.pdf]

Comprehensive genomic profiling of over 10,000 advanced solid tumors

SUPPLEMENTARY MATERIALS

Supplementary Table 1: Distribution of samples with therapeutically relevant alterations, by alteration type and tumor type

	SNV	CNV Amplification	CNV Deletion	Indel	Alt Transcript	Fusion	MSI-H	TMB-H
Total (N = 11091)	9459 (85.29%)	2242 (20.21%)	731 (6.59%)	673 (6.07%)	64 (0.58%)	428 (3.86%)	341 (3.07%)	782 (7.05%)
Anus (N = 38)	27 (71.05%)	9 (23.68%)	5 (13.16%)	4 (10.53%)	0 (0.00%)	1 (2.63%)	0 (0.00%)	1 (2.63%)
Appendix (N = 65)	61 (93.85%)	5 (7.69%)	1 (1.54%)	0 (0.00%)	0 (0.00%)	0 (0.00%)	0 (0.00%)	0 (0.00%)
Biliary (N = 134)	121 (90.30%)	23 (17.16%)	6 (4.48%)	4 (2.99%)	0 (0.00%)	14 (10.45%)	3 (2.24%)	3 (2.24%)
CNS (N = 377)	281 (74.54%)	126 (33.42%)	72 (19.10%)	23 (6.10%)	15 (3.98%)	39 (10.34%)	2 (0.53%)	8 (2.12%)
Breast (N = 2615)	2194 (83.90%)	948 (36.25%)	108 (4.13%)	155 (5.93%)	3 (0.11%)	95 (3.63%)	8 (0.31%)	49 (1.87%)
CRC (N = 1670)	1652 (98.92%)	203 (12.16%)	39 (2.34%)	71 (4.25%)	1 (0.06%)	26 (1.56%)	129 (7.72%)	145 (8.68%)
CUP (N = 127)	115 (90.55%)	21 (16.54%)	8 (6.30%)	5 (3.94%)	1 (0.79%)	5 (3.94%)	6 (4.72%)	13 (10.24%)
Cervix (N = 106)	91 (85.85%)	11 (10.38%)	6 (5.66%)	7 (6.60%)	0 (0.00%)	0 (0.00%)	0 (0.00%)	9 (8.49%)
EOC (N = 528)	486 (92.05%)	87 (16.48%)	41 (7.77%)	27 (5.11%)	0 (0.00%)	11 (2.08%)	10 (1.89%)	12 (2.27%)
Endometrial (N = 471)	458 (97.24%)	59 (12.53%)	12 (2.55%)	71 (15.07%)	0 (0.00%)	11 (2.34%)	96 (20.38%)	119 (25.27%)
Esophagus (N = 132)	125 (94.70%)	68 (51.52%)	12 (9.09%)	9 (6.82%)	0 (0.00%)	0 (0.00%)	3 (2.27%)	2 (1.52%)
Gallbladder (N = 44)	43 (97.73%)	13 (29.55%)	0 (0.00%)	2 (4.55%)	0 (0.00%)	0 (0.00%)	1 (2.27%)	2 (4.55%)
Gastric/GEJ (N = 302)	261 (86.42%)	87 (28.81%)	10 (3.31%)	18 (5.96%)	1 (0.33%)	6 (1.99%)	34 (11.26%)	32 (10.60%)
Gynecologic (N = 33)	33 (100.00%)	9 (27.27%)	1 (3.03%)	1 (3.03%)	0 (0.00%)	0 (0.00%)	0 (0.00%)	2 (6.06%)
Head/Neck (N = 245)	199 (81.22%)	53 (21.63%)	19 (7.76%)	11 (4.49%)	0 (0.00%)	15 (6.12%)	1 (0.41%)	17 (6.94%)
Kidney (N = 492)	419 (85.16%)	9 (1.83%)	8 (1.63%)	33 (6.71%)	1 (0.20%)	10 (2.03%)	1 (0.20%)	2 (0.41%)
Liver (N = 89)	67 (75.28%)	8 (8.99%)	7 (7.87%)	4 (4.49%)	0 (0.00%)	1 (1.12%)	1 (1.12%)	3 (3.37%)
Melanoma (N = 239)	227 (94.98%)	30 (12.55%)	45 (18.83%)	14 (5.86%)	0 (0.00%)	6 (2.51%)	1 (0.42%)	83 (34.73%)
Mesothelioma (N = 30)	24 (80.00%)	0 (0.00%)	2 (6.67%)	1 (3.33%)	0 (0.00%)	0 (0.00%)	0 (0.00%)	2 (6.67%)

NSCLC ($N = 577$)	531 (92.03%)	105 (18.20%)	33 (5.72%)	78 (13.52%)	14 (2.43%)	31 (5.37%)	0 (0.00%)	83 (14.38%)
Neuroblastoma ($N = 45$)	33 (73.33%)	14 (31.11%)	6 (13.33%)	0 (0.00%)	0 (0.00%)	4 (8.89%)	0 (0.00%)	0 (0.00%)
Neuroendocrine ($N = 174$)	111 (63.79%)	16 (9.20%)	25 (14.37%)	6 (3.45%)	0 (0.00%)	2 (1.15%)	1 (0.57%)	9 (5.17%)
Other ($N = 137$)	96 (70.07%)	14 (10.22%)	11 (8.03%)	12 (8.76%)	0 (0.00%)	7 (5.11%)	3 (2.19%)	13 (9.49%)
Pancreas ($N = 478$)	454 (94.98%)	38 (7.95%)	40 (8.37%)	20 (4.18%)	1 (0.21%)	21 (4.39%)	3 (0.63%)	10 (2.09%)
Prostate ($N = 810$)	467 (57.65%)	49 (6.05%)	53 (6.54%)	22 (2.72%)	26 (3.21%)	11 (1.36%)	18 (2.22%)	28 (3.46%)
SCLC ($N = 31$)	29 (93.55%)	8 (25.81%)	5 (16.13%)	1 (3.23%)	0 (0.00%)	0 (0.00%)	0 (0.00%)	4 (12.90%)
Sarcoma ($N = 417$)	239 (57.31%)	97 (23.26%)	82 (19.66%)	19 (4.56%)	1 (0.24%)	78 (18.71%)	9 (2.16%)	18 (4.32%)
Sarcoma-GIST ($N = 52$)	35 (67.31%)	3 (5.77%)	13 (25.00%)	31 (59.62%)	0 (0.00%)	0 (0.00%)	0 (0.00%)	0 (0.00%)
Skin ($N = 71$)	69 (97.18%)	7 (9.86%)	3 (4.23%)	8 (11.27%)	0 (0.00%)	1 (1.41%)	0 (0.00%)	52 (73.24%)
Small Bowel ($N = 81$)	78 (96.30%)	15 (18.52%)	2 (2.47%)	7 (8.64%)	0 (0.00%)	5 (6.17%)	8 (9.88%)	9 (11.11%)
Thymus ($N = 22$)	17 (77.27%)	3 (13.64%)	5 (22.73%)	0 (0.00%)	0 (0.00%)	0 (0.00%)	0 (0.00%)	1 (4.55%)
Thyroid ($N = 160$)	138 (86.25%)	2 (1.25%)	5 (3.12%)	0 (0.00%)	0 (0.00%)	17 (10.62%)	0 (0.00%)	0 (0.00%)
Urothelial ($N = 299$)	278 (92.98%)	102 (34.11%)	46 (15.38%)	9 (3.01%)	0 (0.00%)	11 (3.68%)	3 (1.00%)	51 (17.06%)

Entries show count (percentage). Percentages calculated using tumor-specific sample counts.

Supplementary Table 2: Distribution of samples by therapeutically actionable biomarker and alteration type

Biomarker	Sample count (N)	SNV	CNV Amplification	CNV Deletion	Indel	Alternate transcript	Fusion
TP53	4,948	4809 (97.19%)	0 (0.00%)	37 (0.75%)	115 (2.32%)	0 (0.00%)	0 (0.00%)
PIK3CA	2,017	1962 (97.27%)	16 (0.79%)	0 (0.00%)	54 (2.68%)	0 (0.00%)	0 (0.00%)
KRAS	1,804	1723 (95.51%)	90 (4.99%)	0 (0.00%)	4 (0.22%)	0 (0.00%)	0 (0.00%)
APC	1,515	1509 (99.60%)	0 (0.00%)	4 (0.26%)	4 (0.26%)	0 (0.00%)	0 (0.00%)
PTEN	928	814 (87.72%)	0 (0.00%)	97 (10.45%)	26 (2.80%)	0 (0.00%)	0 (0.00%)
ARID1A	927	919 (99.14%)	0 (0.00%)	2 (0.22%)	13 (1.40%)	0 (0.00%)	0 (0.00%)
CDKN2A	730	369 (50.55%)	0 (0.00%)	348 (47.67%)	15 (2.05%)	0 (0.00%)	0 (0.00%)
ERBB2	601	187 (31.11%)	417 (69.38%)	0 (0.00%)	23 (3.83%)	0 (0.00%)	5 (0.83%)
CCND1	501	20 (3.99%)	481 (96.01%)	0 (0.00%)	0 (0.00%)	0 (0.00%)	0 (0.00%)
BRAF	449	405 (90.20%)	4 (0.89%)	0 (0.00%)	8 (1.78%)	0 (0.00%)	36 (8.02%)
FGF19	409	0 (0.00%)	409 (100.00%)	0 (0.00%)	0 (0.00%)	0 (0.00%)	0 (0.00%)
BRCA2	385	363 (94.29%)	0 (0.00%)	16 (4.16%)	7 (1.82%)	0 (0.00%)	0 (0.00%)
FBXW7	365	361 (98.90%)	0 (0.00%)	3 (0.82%)	1 (0.27%)	0 (0.00%)	0 (0.00%)
RB1	356	320 (89.89%)	0 (0.00%)	35 (9.83%)	4 (1.12%)	0 (0.00%)	0 (0.00%)
CTNNB1	342	325 (95.03%)	2 (0.58%)	1 (0.29%)	14 (4.09%)	0 (0.00%)	0 (0.00%)
FGF3	336	0 (0.00%)	336 (100.00%)	0 (0.00%)	0 (0.00%)	0 (0.00%)	0 (0.00%)
FGF4	331	0 (0.00%)	331 (100.00%)	0 (0.00%)	0 (0.00%)	0 (0.00%)	0 (0.00%)

CDKN2B	324	0 (0.00%)	0 (0.00%)	324 (100.00%)	0 (0.00%)	0 (0.00%)	0 (0.00%)
NF1	324	301 (92.90%)	0 (0.00%)	15 (4.63%)	14 (4.32%)	0 (0.00%)	0 (0.00%)
ATM	311	303 (97.43%)	0 (0.00%)	7 (2.25%)	1 (0.32%)	0 (0.00%)	0 (0.00%)
EGFR	303	136 (44.88%)	134 (44.22%)	1 (0.33%)	64 (21.12%)	19 (6.27%)	6 (1.98%)
PBRM1	299	286 (95.65%)	0 (0.00%)	4 (1.34%)	9 (3.01%)	0 (0.00%)	0 (0.00%)
MAP3K1	273	260 (95.24%)	0 (0.00%)	9 (3.30%)	10 (3.66%)	0 (0.00%)	0 (0.00%)
PIK3R1	273	185 (67.77%)	0 (0.00%)	7 (2.56%)	92 (33.70%)	0 (0.00%)	0 (0.00%)
RNF43	242	240 (99.17%)	0 (0.00%)	0 (0.00%)	2 (0.83%)	0 (0.00%)	0 (0.00%)
MDM2	218	0 (0.00%)	218 (100.00%)	0 (0.00%)	0 (0.00%)	0 (0.00%)	0 (0.00%)
MYC	218	1 (0.46%)	217 (99.54%)	0 (0.00%)	0 (0.00%)	0 (0.00%)	0 (0.00%)
ASXL1	210	208 (99.05%)	0 (0.00%)	0 (0.00%)	2 (0.95%)	0 (0.00%)	0 (0.00%)
VHL	208	194 (93.27%)	0 (0.00%)	1 (0.48%)	13 (6.25%)	0 (0.00%)	0 (0.00%)
SETD2	206	200 (97.09%)	0 (0.00%)	0 (0.00%)	6 (2.91%)	0 (0.00%)	0 (0.00%)
PRKDC	205	196 (95.61%)	2 (0.98%)	2 (0.98%)	4 (1.95%)	0 (0.00%)	1 (0.49%)
ATRX	204	186 (91.18%)	0 (0.00%)	15 (7.35%)	3 (1.47%)	0 (0.00%)	0 (0.00%)
FGFR1	201	20 (9.95%)	171 (85.07%)	0 (0.00%)	0 (0.00%)	0 (0.00%)	18 (8.96%)
KDM6A	201	185 (92.04%)	0 (0.00%)	14 (6.97%)	2 (1.00%)	0 (0.00%)	0 (0.00%)
BRCA1	200	190 (95.00%)	0 (0.00%)	3 (1.50%)	7 (3.50%)	0 (0.00%)	0 (0.00%)
AKT1	198	193 (97.47%)	8 (4.04%)	0 (0.00%)	0 (0.00%)	0 (0.00%)	0 (0.00%)
NRAS	195	195 (100.00%)	0 (0.00%)	0 (0.00%)	0 (0.00%)	0 (0.00%)	0 (0.00%)
ESR1	190	119 (62.63%)	13 (6.84%)	0 (0.00%)	0 (0.00%)	0 (0.00%)	71 (37.37%)
ARID2	180	173 (96.11%)	0 (0.00%)	2 (1.11%)	5 (2.78%)	0 (0.00%)	0 (0.00%)
MSH6	175	169 (96.57%)	0 (0.00%)	1 (0.57%)	5 (2.86%)	0 (0.00%)	0 (0.00%)
MTAP	166	0 (0.00%)	0 (0.00%)	166 (100.00%)	0 (0.00%)	0 (0.00%)	0 (0.00%)
CREBBP	159	144 (90.57%)	0 (0.00%)	3 (1.89%)	12 (7.55%)	0 (0.00%)	1 (0.63%)
BAP1	157	143 (91.08%)	0 (0.00%)	10 (6.37%)	5 (3.18%)	0 (0.00%)	0 (0.00%)
FGFR2	157	88 (56.05%)	44 (28.03%)	0 (0.00%)	0 (0.00%)	0 (0.00%)	36 (22.93%)
MSH3	152	149 (98.03%)	0 (0.00%)	1 (0.66%)	2 (1.32%)	0 (0.00%)	0 (0.00%)
SMARCA4	152	141 (92.76%)	0 (0.00%)	3 (1.97%)	9 (5.92%)	0 (0.00%)	0 (0.00%)
CCNE1	143	0 (0.00%)	143 (100.00%)	0 (0.00%)	0 (0.00%)	0 (0.00%)	0 (0.00%)
GNAS	131	130 (99.24%)	1 (0.76%)	0 (0.00%)	0 (0.00%)	0 (0.00%)	0 (0.00%)
STK11	129	107 (82.95%)	0 (0.00%)	19 (14.73%)	3 (2.33%)	0 (0.00%)	0 (0.00%)
MCL1	127	0 (0.00%)	127 (100.00%)	0 (0.00%)	0 (0.00%)	0 (0.00%)	0 (0.00%)
MAP2K4	126	105 (83.33%)	0 (0.00%)	20 (15.87%)	1 (0.79%)	0 (0.00%)	0 (0.00%)
CDK4	122	5 (4.10%)	117 (95.90%)	0 (0.00%)	0 (0.00%)	0 (0.00%)	0 (0.00%)
POLE	122	118 (96.72%)	0 (0.00%)	2 (1.64%)	3 (2.46%)	0 (0.00%)	0 (0.00%)
ERBB3	116	108 (93.10%)	7 (6.03%)	0 (0.00%)	0 (0.00%)	0 (0.00%)	1 (0.86%)
EP300	111	102 (91.89%)	0 (0.00%)	2 (1.80%)	8 (7.21%)	0 (0.00%)	0 (0.00%)
ATR	107	106 (99.07%)	0 (0.00%)	0 (0.00%)	1 (0.93%)	0 (0.00%)	0 (0.00%)
TSC1	107	101 (94.39%)	0 (0.00%)	1 (0.93%)	5 (4.67%)	0 (0.00%)	0 (0.00%)
FGFR3	106	79 (74.53%)	10 (9.43%)	0 (0.00%)	0 (0.00%)	0 (0.00%)	19 (17.92%)
KIT	104	40 (38.46%)	40 (38.46%)	0 (0.00%)	34 (32.69%)	0 (0.00%)	0 (0.00%)
MTOR	104	103 (99.04%)	0 (0.00%)	0 (0.00%)	1 (0.96%)	0 (0.00%)	0 (0.00%)
KDM5C	103	93 (90.29%)	0 (0.00%)	6 (5.83%)	4 (3.88%)	0 (0.00%)	0 (0.00%)
STAG2	103	89 (86.41%)	0 (0.00%)	9 (8.74%)	5 (4.85%)	0 (0.00%)	0 (0.00%)

Biomarker alteration frequencies provided at the sample-level; row percentages calculated at the sample-level

Supplementary Table 3: Distribution of samples with alternate transcripts, overall and by tumor type

**Tumor Type/*Gene***	**Frequency (%)**
Total (*N* = 10,227)	
ARv7	63 (0.6%)
EGFRvIII	15 (0.1%)
EGFRvIVa	2 (0.0%)
EGFRvIVb	2 (0.0%)
METe14	18 (0.2%)
**CNS (*N* = 344)**	
EGFRvIII	12 (3.5%)
EGFRvIVb	2 (0.6%)
METe14	1 (0.3%)
**Breast (*N* = 2,445)**	
ARv7	35 (1.4%)
METe14	2 (0.1%)
**CRC (*N* = 1,555)**	
EGFRvIVa	1 (0.1%)
**CUP (*N* = 110)**	
METe14	1 (0.9%)
**Gastric/GEJ (*N* = 278)**	
EGFRvIII	1 (0.4%)
Head/Neck (*N* = 222)	
ARv7	2 (0.9%)
**Kidney (*N* = 436)**	
EGFRvIII	1 (0.2%)
**NSCLC (*N* = 523)**	
METe14	14 (2.7%)
**Pancreas (*N* = 412)**	
EGFRvIVa	1 (0.2%)
**Prostate (*N* = 748)**	
ARv7	26 (3.5%)
**Sarcoma (*N* = 394)**	
EGFRvIII	1 (0.3%)

Analysis limited to samples with both DNA and RNA sequencing

Supplementary Table 4: Biomarkers included in the cancer-relevant pathways

Pathways	Biomarkers
Phosphoinositide 3-kinase/ Protein kinase B/mammalian target of rapamycin (PI3K/ AKT/mTOR)	*PIK3CA, AKT1, AKT2, AKT3, PIK3CB, MTOR, PTEN, RICTOR, RPTOR, PIK3CD, TSC1, TSC2, STK11, PIK3R1, PIK3R2*
Mitogen-activated protein kinase (MAPK)	*KRAS, NRAS, HRAS, BRAF, MAP2K1, MAP2K7, MAP2K2, MAP2K4, MAP3K1, MAP4K2, MAP4K3, MAP3K8, RASGRF1, NF1, RAF1, NF2, RASA1, ARAF*
DNA damage response	*ARID1A, ATM, ATR, ATRX, BAP1, BARD1, BLM, BRCA1, BRCA2, BRIP1, CDK12, CHEK1, CHEK2, EPCAM, ERCC1, ERCC2, ERCC3, ERCC4, ERCC5, FANCA, FANCC, FANCD2, FANCE, FANCF, FANCG, FANCI, FANCL, FANCM, MRE11A, MUTYH, NBN, PALB2, PPP2R2A, RAD21, RAD50, RAD51, RAD51B, RAD51C, RAD51D, RAD52, RAD54L, XRCC1, XRCC2, XRCC3*
Immuno-oncology	*POLE, POLD1, PMS2, PMS1, PDCD1LG2, PBRM1, MSH6, MSH3, MSH2, MLH1, CTLA4, CD274, ARID2*, MSI-High, TMB-High
Cell cycle	*CCND1, CCND2, CCND3, CDK4, CDK6, RB1, E2F1, E2F3, SMAD2, SMAD3, SMAD4, CDKN2A, CDKN2B, CDKN2C, CDKN1B, CDKN1A, CCNE1, CDC6, CDK1, TP53, CREBBP, EP300, PRKDC, MDM2*
Receptor tyrosine kinase (RTK)	*ALK, EGFR, ERBB2, FGFR1, FGFR2, FGFR3, KIT, MET, PDGFRA, PDGFRB, RET, ROS1*
